# Rheumatoid Meningitis Presenting Without Apparent Joint Symptoms: A Case Report Highlighting the Usefulness of Musculoskeletal Ultrasound

**DOI:** 10.7759/cureus.95629

**Published:** 2025-10-28

**Authors:** Yuta Honkawa, Yusuke Nakazawa, Wataru Shiraishi

**Affiliations:** 1 Neurology, Kokura Memorial Hospital, Kitakyushu, JPN; 2 Interventional Neuroradiology, Kokura Memorial Hospital, Kitakyushu, JPN; 3 Internal Medicine, Shiraishi Internal Medicine Clinic, Nogata, JPN

**Keywords:** anti-cyclic citrullinated peptide antibody index, cerebrospinal fluid (csf), diagnostic musculoskeletal ultrasound, magnetic resonance imaging, rheumatoid arthritis, rheumatoid meningitis

## Abstract

Rheumatoid meningitis (RM) is a rare central nervous system complication of rheumatoid arthritis. Symptoms of RM are diverse, including headache, convulsions, hemiplegia, movement disorders, cognitive decline, and consciousness disorders. Sometimes, RM can present neurological symptoms prior to joint symptoms, which can be a diagnostic challenge. Here, we report a case of RM without obvious arthritis symptoms, which was later diagnosed as RM supported by musculoskeletal ultrasound findings. The diagnosis of RM was made based on characteristic brain MRI findings, positive anti-cyclic citrullinated peptide antibodies (ACPA) in both serum and cerebrospinal fluid, an elevated ACPA index, and exclusion of infections and malignancy. Treatment with high-dose intravenous corticosteroid led to rapid clinical improvement. Our case highlights the diagnostic utility of musculoskeletal ultrasound in detecting subclinical arthritis and suggests its potential role in evaluating suspected RM even in the absence of joint symptoms.

## Introduction

Rheumatoid meningitis (RM) is a rare extra-articular manifestation of rheumatoid arthritis (RA), occurring in less than 1% of RA patients [1]. It often presents a variety of neurological symptoms, including headaches, seizures, altered mental status, movement disorders, and focal deficits, which sometimes mimic strokes [2]. RM can occur independently of RA disease activity or duration, and diagnosis may be difficult in patients without a prior history of RA or obvious joint symptoms [3].

The classification criteria for RA include the number of joints involved [4], which refers to joints showing clinical signs of inflammation, such as tenderness or swelling. However, even in patients considered to be in clinical remission, subclinical synovitis with a positive power Doppler signal can be detected by musculoskeletal ultrasound (MSUS). Previous studies have shown that such findings are significantly associated with RA activity [5]. These observations suggest that MSUS is a valuable tool for assessing disease activity in RA, even in the absence of overt joint symptoms. Here, we report a case of RM in a 77-year-old woman with no apparent joint symptoms, where MSUS played a pivotal role in confirming the diagnosis of RA.

## Case presentation

A 77-year-old Japanese woman was found collapsed in her room at home in the morning and was transported to the emergency department. She had a history of Alzheimer’s dementia and interstitial pneumonia, but was not under active medical care. Although she had mild short-term memory impairment, she had been living independently. She had dinner with her family the night before and returned to her room thereafter.

On arrival, her Glasgow Coma Scale (GCS) score was 11/15 (E3V3M5). Her body temperature was 38.1°C. Blood pressure was 178/102 mmHg without laterality, heart rate was 76 beats per minute and regular, and peripheral oxygen saturation was 99% under 1 L/min of supplemental nasal oxygen. On physical examination, there were no apparent joint symptoms or pain.

Neurological examination revealed pupils 3 mm in diameter bilaterally with preserved light reflexes. She exhibited left-sided myoclonic jerks without voluntary movement, whereas voluntary movement was observed in the right upper and lower extremities. The Babinski sign was positive with an extensor plantar response on the left. Deep tendon reflexes were symmetric.

Laboratory findings showed a white blood cell count of 13,540/μL, C-reactive protein (CRP) level of 12.1 mg/dL, and rheumatoid factor of 59 IU/mL (Table 1). Cerebrospinal fluid (CSF) analysis revealed mild pleocytosis (8 cells/μL; normal < 5 cells/μL), normal protein and glucose levels, and negative oligoclonal bands. Anti-cyclic citrullinated peptide antibodies (ACPA) were markedly elevated in serum (3245 U/mL; normal < 4.5 U/mL) and positive in CSF (66.3 U/L). The ACPA index was 2.7 (normal < 1.3), suggesting intrathecal antibody synthesis. Infectious and neoplastic causes were excluded, as contrast-enhanced CT from the neck to pelvis revealed no malignancy, and all cultures of blood, urine, and CSF were negative. No other autoimmune abnormalities were identified.

**Table 1:** Results of laboratory tests ACPA: anti-cyclic citrullinated peptide antibody; CRP: C-reactive protein; CSF: cerebrospinal fluid; ESR: erythrocyte sedimentation rate; IgG: immunoglobulin G; MNCs: mononuclear cells; PMNs: polymorphonuclear leukocytes; RF: rheumatoid factor; WBC: white blood cell

	Laboratory Parameters	Patient Value	Reference Range
Serum	WBC	13540 /μL	3000-8900 /μL
	Neutrophils	85.8%	44-73%
	Glucose	131 mg/dL	70-109 mg/dL
	IgG	1199 mg/dL	870-1700 mg/dL
	CRP	12.1 mg/dL	<0.5 mg/dL
	ESR (1h)	35 mm	3-15 mm
	Treponema pallidum antibody	negative	negative
	β-D glucan	4.66 pg/mL	<20.0 pg/mL
	T-SPOT. TB	negative	negative
	ACPA	3245 U/mL	<4.5 U/mL
	RF	59 IU/mL	<15 IU/mL
	Antinuclear antibody	negative	negative
	Anti-SS-A antibody	negative	negative
	Anti-SS-B antibody	negative	negative
	Anti-neutrophil cytoplastic antibodies	negative	negative
	Soluble interleukin-2 receptor	950 U/mL	122-496 U/mL
	Matrix metalloproteinase-3	19.8 ng/mL	17.3-59.7 ng/mL
	Angiotensin-coverting enzyme	9.3 U/L	7.0-25.0 U/L
CSF	Color	Clear	-
	WBC	8 /μL	0-3 /μL
	PMNs	6 /μL	-
	mononuclear cells	2 /μL	-
	Protein	37 mg/dL	15-45 mg/dL
	Glucose	82 mg/dL	-
	IgG	9.1 mg/dL	1-4 mg/dL
	ACPA	66.3 U/mL	-
	ACPA index	2.69	<1.3

**Figure 1:**
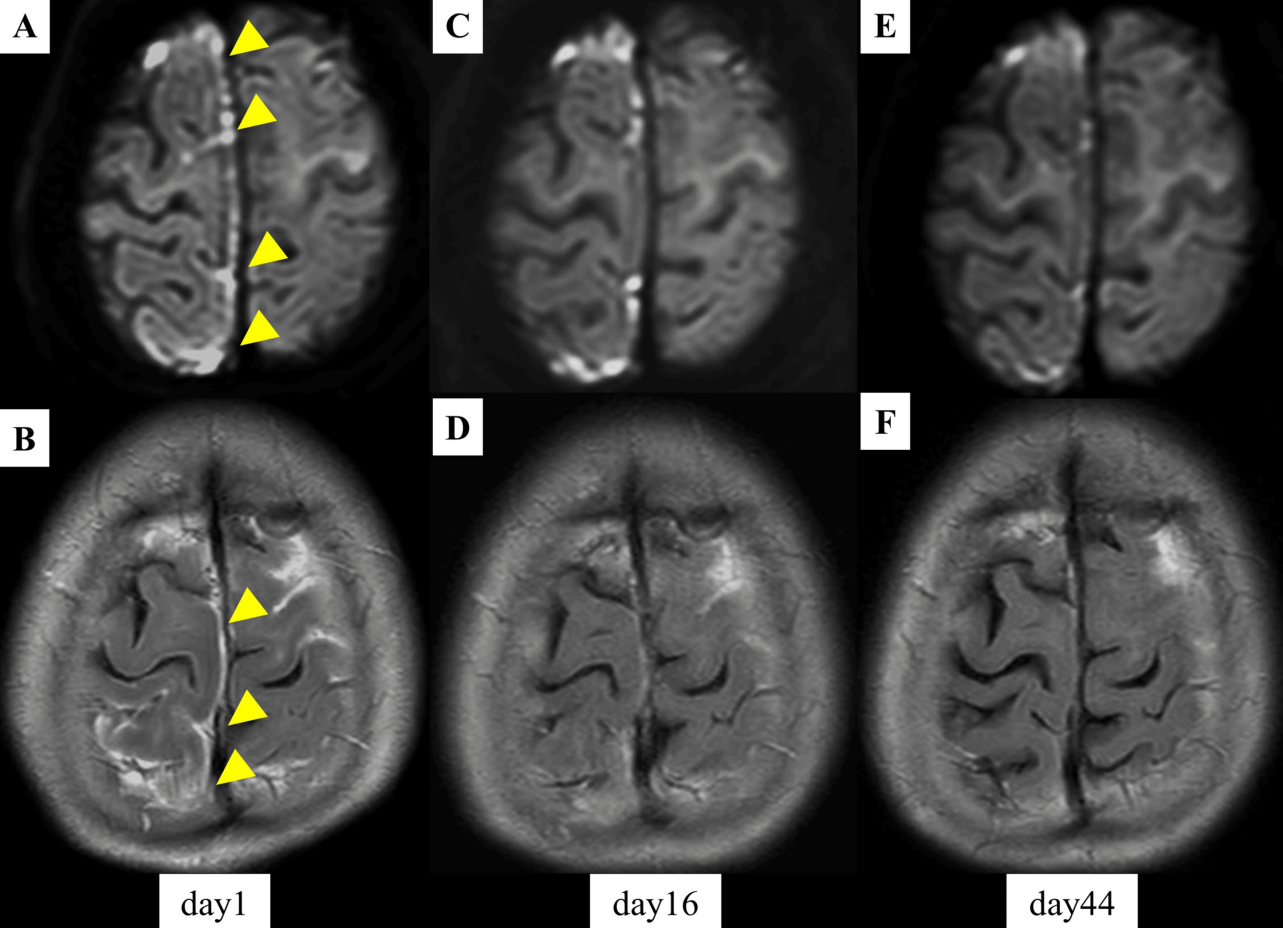
Brain MRI findings of the patient MRI findings on day 1 (A, B), day 16 (C, D), and day 44 (E, F). The upper row (A, C, E) shows diffusion-weighted imaging (DWI), and the lower row (B, D, F) shows gadolinium contrast-enhanced fluid-attenuated inversion recovery (FLAIR) images. Right-predominant cortical and brain sulcus lesions with contrast enhancement (arrow heads) gradually improved over time.

Brain MRI showed no parenchymal abnormalities on conventional sequences. However, gadolinium-enhanced fluid-attenuated inversion recovery (FLAIR) imaging revealed hyperintense lesions with leptomeningeal enhancement predominantly along the right cerebral convexity (Figure 1). Some of these lesions showed high signal intensity on diffusion-weighted imaging (DWI) with iso-intensity on the apparent diffusion coefficient (ADC) map. No hemorrhagic findings were observed on T2*-weighted imaging or head CT, and magnetic resonance angiography (MRA) revealed no intracranial arterial stenosis or occlusion.

Based on the imaging findings of high-intensity lesions in the cerebral sulci on DWI with gadolinium enhancement and the blood test results, RM was an important differential diagnosis.

**Figure 2:**
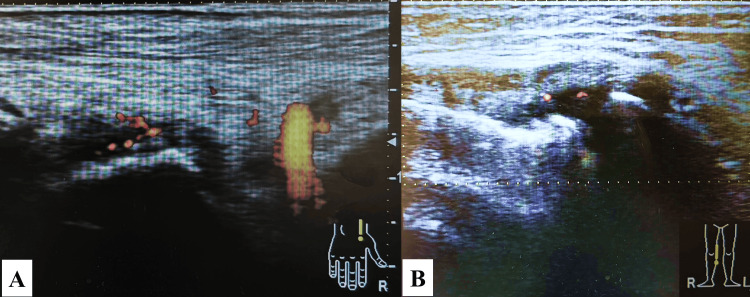
Ultrasonography of right wrist joint (A) and right knee joint (B) Ultrasonography shows synovial thickening with an active Doppler signal (color) indicating synovial inflammation.

Although no apparent joint symptoms were initially observed, re-examination revealed swelling of the right knee and right fifth toe. MSUS demonstrated synovitis in the right knee and wrist with a power Doppler signal corresponding to grade 2 activity. Taken together with the serological markers, she fulfilled the 2010 American College of Rheumatology and European League Against Rheumatism (now called European Alliance of Associations for Rheumatology) (ACR/EULAR) classification criteria for RA [4], leading to a diagnosis of RM (Figure 2).

High-dose intravenous methylprednisolone (1,000 mg/day for three days) was initiated, followed by oral prednisolone tapering. The patient’s neurological status improved markedly to GCS 15/15 (E4V5M6), with resolution of fever and amelioration of the left hemiparesis. Follow-up MRI on days 16 and 44 showed improvement of brain lesions. CSF ACPA index decreased to 0.88 on day 18, indicating treatment response. Oral prednisolone was continued at a maintenance dose of 5 mg/day. The patient was transferred for rehabilitation on day 45 and was referred to the rheumatologist for RA management.

## Discussion

This case illustrates the diagnostic challenge of RM in the absence of preceding joint symptoms. While RM often occurs in patients with longstanding RA, there are increasing reports of cases without joint symptoms at onset. In our literature review, we found 24 RM cases, including our case, without previous joint symptoms reporting (Table 2) [6-26], but no case was diagnosed based on MSUS findings. MSUS, particularly power Doppler imaging, has been reported to be superior to clinical assessment in detecting active inflammation in RA, and is also helpful in predicting relapse or radiographic progression in patients in clinical remission [27]. MSUS is a non-invasive and sensitive method for detecting subclinical synovitis of RA, and its role may be underappreciated in the diagnosis of RM.

**Table 2:** Clinical features of rheumatoid meningitis without arthritis at onset *Medications and dosages at the initiation of maintenance therapy. **Length of time to complete treatment. CPA: cyclophosphamidel; DEX: dexamethasone; F: female; M: male; mPSL: methylprednisolone; MTX: methotrexate; PSL: prednisolone; RA: rheumatoid arthritis; RTX: rituximab; “+” indicates presence; “-” indicates absence; NA: data not available or not reported in the original article; WNL: within normal limits; CRP: C-reactive protein; RF: rheumatoid factor

Study (Authors)	Case	Age (years)	Sex	Fever	Headache	Neurological symtpoms	Blood			CSF			Management		
							CRP (mg/dL)	RF (IU/mL)	ACPA (U/mL)	WBC (/μL)	Protein (mg/dL)	ACPA indx	Acute treatment	Maintenance*	Duration (months)**
Kim et al [6]	1	66	M	NA	NA	Epilepsy, confusion, left paralysis	NA	Increased	1448	11	WNL	NA	-	Oral steroids	6
Kawabata et al. [7]	2	69	F	-	-	Motor apraxia, right paralysis, convulsions	NA	NA	31.4	47	37	NA	mPSL pulse	Oral steroids	NA
Padjen et al. [8]	3	77	F	NA	NA	Right paralysis, convulsions	4.3	172	405	WNL	WNL	NA	PSL	MTX	NA
Abe et al. [9]	4	84	F	-	+	Cognitive impairment, apraxia, left paralysis	WNL	66	1150	7	43	2.46	mPSL pulse	Oral steroids	NA
Magaki et al. [10]	5	37	M	-	+	Right facial weakness, speech difficulty, right hand clumsiness and paresthesia, transient cognitive dysfunction	4.7	83	>250	16	35	NA	PSL	Oral steroids	NA
Shibahara et al. [11]	6	63	M	+	+	Dizziness, confusion	0.4	140	472	37	98	NA	mPSL pulse	Oral steroids	NA
Jessee and Keenan [12]	7	68	F	NA	NA	Confusion, right paralysis, convulsions	NA	208	95.8	8	64	NA	PSL	MTX	NA
Schuster et al. [13]	8	48	M	NA	+	Transient left paralysis	WNL	298	>340	300	137	NA	mPSL pulse	Oral steroids	2
Schuster et al. [13]	9	72	M	NA	NA	Transient left weakness and numbness	WNL	133	154	51	WNL	NA	mPSL pulse	Oral steroids	15
Finkelshtein et al. [14]	10	66	M	NA	+	Transient left lower limb weakness, epileptic seizures	WNL	25	266	WNL	WNL	NA	-	-	-
Lee et al. [15]	11	72	F	NA	NA	Left paralysis, aphasia, mental disorder, epilepsy	0.9	WNL	198	12	25	NA	mPSL pulse	CPA	4
Kira et al. [16]	12	93	M	-	NA	Unconsciousness, epileptic seizures	2	223	306	3	68	NA	Half mPSL pulse	Oral steroids	NA
McKenna et al. [17]	13	59	M	+	+	Left paralysis, seizures	1.8	88.2	>340	NA	672	NA	mPSL pulse	Oral steroids	NA
Yamaoka et al. [18]	14	62	F	+	NA	Abnormal behavior, unconsciousness, seizures, cognitive dysfunction	WNL	75	30.7	WNL	121	1.5	mPSL pulse	Oral steroids	2
Iwao et al. [19]	15	71	M	+	+	Right lower limb weakness, gait disturbance	0.37	17.9	115	35.7	98.8	9	-	Oral steroids	NA
Rodriguez et al. [20]	16	62	M	+	+	Right lower limb weakness	NA	579	>150	Increased	27.7	NA	DEX pulse	Oral steroids	NA
Chouk et al. [21]	17	62	F	NA	+	Sudden unusual sensations, seizures	NA	90	340	20	51	NA	mPSL pulse	RTX	12
Matsuda et al. [22]	18	77	M	-	-	Gait disturbance, communication difficulties	0.67	29	173	60	116	6.4	mPSL pulse	Oral steroids	NA
Yang et al. [23]	19	80	F	NA	NA	Transient left paralysis	2.85	WNL	>200	15	78	NA	-	-	-
Yang et al. [23]	20	65	M	-	-	Inability to close the left eye, left-side headache	NA	WNL	WNL	WNL	WNL	NA	PSL	-	-
Muramatsu et al. [24]	21	75	M	-	-	Paraplegia, left lower limb convulsions	0.85	16	238	25/3	68	6	mPSL pulse	Oral steroids	5
Murakami et al. [25]	22	32	F	-	-	Epileptic seizures	NA	47	578	49	56	7.2	mPSL pulse	Oral steroids	NA
Kita et al. [26]	23	52	F	-	+	Unconsciousness, communication difficulties	4.73	20	1170	55	108	7.3	mPSL pulse	Oral steroids	13
Our case	24	77	F	+	-	Epileptic seizures, cognitive impairment	12.1	59	3245	8	37	2.7	mPSL pulse	Oral steroids	5

Brain biopsy can aid diagnosis, particularly when ACPA is not elevated, but it is invasive and not always feasible, especially in elderly patients. In our case, a biopsy was not performed due to the patient's age and clinical risks. Instead, diagnosis was based on characteristic MRI findings, elevated ACPA and ACPA index, MSUS results, and exclusion of other etiologies. Clinical improvement following corticosteroid therapy further supported the diagnosis of RM.

There is no consensus on maintenance therapy for RM, but most cases respond well to corticosteroids. In our patient, oral prednisolone was continued after the initial intravenous therapy, and RA treatment was initiated under rheumatologic care.

## Conclusions

We described a rare case of RM presenting without preceding joint symptoms, the diagnosis of which was supported by non-invasive MSUS. Even in the absence of apparent arthritis symptoms, MSUS may reveal subclinical joint inflammation and support the diagnosis of RA, which in turn is critical for diagnosing RM. This case underscores the importance of a comprehensive and multimodal approach, including imaging, serology, and ultrasound, in evaluating patients with unexplained neurological symptoms and possible RM.
